# Emphasising Organisational Routine: A Qualitative Study of Patient and Health Professional Experiences of Inpatient Oncology Care

**DOI:** 10.3390/healthcare10112145

**Published:** 2022-10-28

**Authors:** Jennifer Watermeyer, Harriet Etheredge, June Fabian, Sue Tager

**Affiliations:** 1 Health Communication Research Unit, School of Human and Community Development, University of the Witwatersrand, 1 Jan Smuts Ave., Braamfontein, Johannesburg 2000, South Africa; 2 Wits Donald Gordon Medical Centre, School of Clinical Medicine, University of the Witwatersrand, 21 Eton Rd, Parktown, Johannesburg 2193, South Africa; 3 Steve Biko Centre for Bioethics, School of Clinical Medicine, University of the Witwatersrand, 29 Carse O’Gowrie Rd, Parktown, Johannesburg 2193, South Africa

**Keywords:** cancer, communication, experience, hospitals, inpatients, qualitative research, oncologists, psycho-oncology, patient care, uncertainty

## Abstract

Background: Although the experience of hospitalisation for cancer management has been widely researched, such research from the African sub-continent is limited. Objective: This study explored experiences of patient care in a tertiary, inpatient oncology setting in urban South Africa, from the point of view of patients and health professionals. Methods: In-depth interviews and focus groups were conducted with participants. Participants included oncology inpatients, oncologists, nurses and nursing management (N = 46) at an oncology unit in Johannesburg, South Africa. Data were analysed by a multidisciplinary research group using reflexive thematic analysis principles. Results: Our results suggest that barriers to establishing effective organisational routines included communication breakdowns between patients and healthcare providers, a lack of predictability in interactions with doctors, deficient access to information and diminished confidence in nurses. Conclusions: Oncology inpatients may not feel in control of their circumstances, in part due to lacking routine in the hospital setting. Ironically, nurses, who are often at the frontline of patient management, appear to be underutilised or disabled by the healthcare system as conveyors of information. Implications for practice: Robust organisational routines for oncology inpatients may be a good mechanism for allaying uncertainty and conferring a sense of control. Nursing staff, as the individuals with the most direct patient contact, could be instrumental in nurturing organisational routines towards improving patient perceptions of care.

## 1. Introduction

### Background to the Study

Cancers constitute the highest burden of disease in both men and women worldwide [1]. Alongside cardiovascular disease and respiratory disease, cancer management has been given global priority by the United Nations [2]. Whilst it accounts for the highest number of deaths reported in the Americas and the second-highest number of deaths in Europe, cancer is the fourth most common cause of death in Africa [1]. This may be due to underdiagnosis and the irregular reporting of cancer-related mortality across Africa, and could also be explained by a dual burden of persistent infectious diseases (malaria, HIV and TB) coupled with emerging non-communicable diseases. Additionally, non-communicable diseases such as renal and cardiovascular disease and diabetes mellitus are associated with relatively high mortality in Africa, compared to high-income settings [3,4].

The diagnosis, treatment and ongoing management of cancer can be a lengthy and resource-intensive process. Regardless of context or setting, the optimal management of oncology patients requires an integrated horizontal multidisciplinary team approach [2]. This approach begins with mutually agreed upon individualised treatment plans, followed by ensuring each aspect of care is appropriately administered within a broader framework of ongoing review and support [5]. This creates a sense of control during the care pathway for patients and their families, presupposing that some kind of routine or plan is in place.

Multidisciplinary team models of care are challenging in traditionally siloed healthcare systems, and effective communication is critical to their success. Individuals within the healthcare team are required to communicate with each other and with the patient (and family or caregivers) across the continuum of care [6]. This means that good communication needs to supersede physical and temporal barriers, and navigate the complexity created through the discipline-specific nature of different practices. With ever-increasing numbers of people involved in the chain of oncological care, opportunities for communication breakdowns arise that can negatively affect patient perceptions of care.

The experience of hospitalisation for cancer management has been widely researched internationally [7,8]. Across hospitalised oncology study populations, disempowerment in the hospital system is an ongoing theme. In addition, inpatient experiences may be frustrated by a lack of access to clinical trials and the perceived inexperience of staff in managing patients [7]. Access (or lack thereof) to information may add complexity to the patient experience, especially information that is only available from health professionals [9]. In some studies, experiences of cancer care are reportedly more positive, with some patients indicating the hospital became “a second home” to them over time [8]. Few studies triangulate the experience of oncology inpatients and their families with those of the healthcare team involved in their care [7,8].

## 2. Materials and Methods

We conducted a qualitative study based on phenomenological principles. Qualitative methods were chosen for this study as we sought to better understand experiences and perceptions, which cannot always be quantified. Three of the authors are themselves healthcare workers, and one of the authors is a medical bioethicist.

The study took place in the oncology department of an academic hospital in South Africa. The study was approved by the local Institutional Review Board, and all participants provided written consent. All participants were over the age of 18. Participants were approached on a face-to-face basis and invited to participate in the study. Data were collected by Author 2, a medical bioethicist. No prior relationships with any participants had been established.

**Figure 1:**
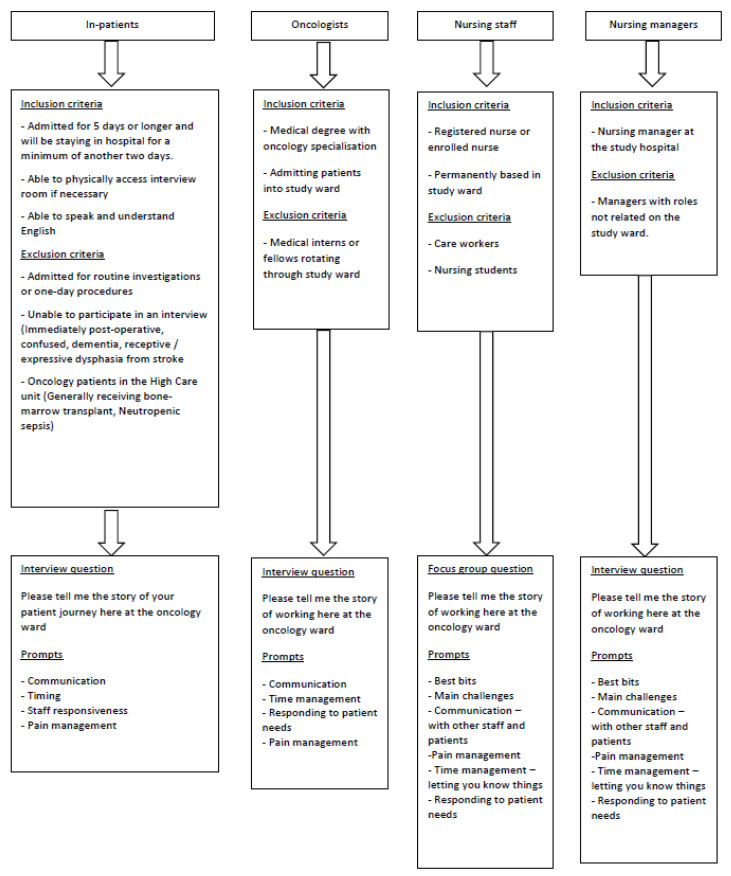
Sampling strategy.

In-depth interviews were undertaken with inpatients and oncologists and focus groups with nurses and nurse managers. A purposive sampling strategy was used, and the inclusion and exclusion criteria for each group are detailed in Figure 1. Although we had planned to conduct interviews for all participant groups, the health professionals’ shifts and schedules necessitated the use of focus groups instead. Focus group participants were recruited by the PI and invited to participate in the study. All were made aware that confidentiality in a focus group cannot be guaranteed.

Participants included twenty-five oncology inpatients, four oncologists, fourteen nurses, and three nurse managers (N = 46). Data were collected over a period of six months. Data collection was concluded once the research team agreed data saturation had been reached across each sample group and in the triangulated data corpus. One patient refused to participate due to a complex spousal relationship. Four oncologists and one member of the nursing staff declined to participate due to work and time commitments. All management staff invited to participate agreed.

Following a narrative approach, we asked one main question related to participants’ experiences of the oncology ward (their ‘story’ of the ward) which was adjusted according to the role of each participant (refer to Figure 1). Patients, staff and management led us in formulating the research questions and refining the methods used through participation in pilot studies and informal interactions to gauge some of the issues that were deemed important to explore.

Participants were informed that the objective of the study was to better understand perceptions of patient care. Interactions took place in a private room, with only participants and the PI present. Interviews were audio recorded and transcribed by the PI, and the average length of interactions was 50 min. Interviews were conducted in English.

Data were analysed by a multidisciplinary research group using reflexive thematic analysis principles [10]. The team met four times in total, twice during the data collection phase and twice after data collection was complete. The data analysis team included two medical doctors, a nursing sister, a medical historian, a bioethicist, a musician with experience working in hospital spaces and three experts in medical humanities. Codes and themes were identified, refined, defined and named by the research team. This was done by supplying the transcripts to the team ahead of time, and each individual coded every transcript. Codes and themes were then agreed upon during the face-to-face meetings of the analysis team.

Trustworthiness and rigour were safeguarded through reflective journaling, peer debrief, member checking and ensuring transcription reliability. COREQ guidelines were used. The substantial sample size and diverse study population lends to transferability and credibility. The use of different data collection techniques and the triangulation of data from different sources also enhances credibility. Great caution has been taken to protect the identities of the study participants.

Results were shared with all participants in different formats. Patients were offered direct feedback by the researcher. An infographic was created for medical doctors, and feedback to the nursing staff was done during three lunch-hour sessions that allowed for informal discussion of the main findings. Nurses were also given an infographic to lead discussions.

The study was approved by the University of the Witwatersrand Human Research Ethics Committee (Medical)—Clearance number M150218. During the interview and analysis process, it became clear that the study results were not only highly sensitive but could also potentially identify participants. We have taken great care, in presenting the results, to ensure that the highest ethical standards are met, and that the confidentiality challenges are very carefully managed.

### Research Gap

The purpose of this study is to explore perceptions of care amongst patients and healthcare workers at a single oncology centre, in Johannesburg, South Africa. Although the experience of hospitalisation for cancer management has been widely researched internationally, there are no publications from the African sub-continent that triangulate data from oncology in-patients and their families with those from medical and nursing staff and hospital management involved in their care. Thus, this study addresses a gap in the literature.

## 3. Results

Thematic analysis yielded 23 codes which were grouped into eight main themes (described in Table 1). These themes overlapped and intersected at times across the three sub-groups of participants. Some additional themes less frequently mentioned by participants related to the South African context in which care was provided, the infrastructure and daily running of the hospital and pain control. These did not constitute the main research findings.

Our findings suggest some alignment but also several mismatches across participant group experiences, and these encompass the diversity of the sample, as well as illustrate the powerful expectations that each group has on the others. Mismatches in experiences were particularly related to organisational routines and communication. Patients expressed the overwhelming need for a sense of routine, to be able to plan their days in the hospital and for genuine agency in the healthcare journey. Interestingly, doctors acknowledged these needs superficially, but barriers to their realisation presented themselves in practice. These include doctors being sometimes unaware of patient perceptions and hindered by their workload (which is often extremely large), resulting in unrecognised or substantially unmet needs in the patient population. Nurses, on the other hand, were perhaps best positioned to meaningfully contribute to patient management, but felt disempowered to do so, despite spending the most time with patients. In general, nurses seemed to feel ‘stuck in the middle’, often lacking the necessary information to give to patients in a way that might cement a sense of routine. Nurses also felt under-skilled and undervalued. This perception was reinforced by both patients and doctors in the sample.

Meanwhile, patients felt a distinct deficiency in access to information regarding their condition and treatment. This access was compromised by the perceived unavailability and lack of communication with doctors in particular and some doubts about the nursing staff as a source of reliable information. Patients felt that when they did see their doctor, there was often insufficient time to ask questions or discuss a treatment plan. Doctors, however, felt that they were communicating efficiently with patients, albeit within severe restrictions of time.

## 4. Discussion

**Figure 2:**
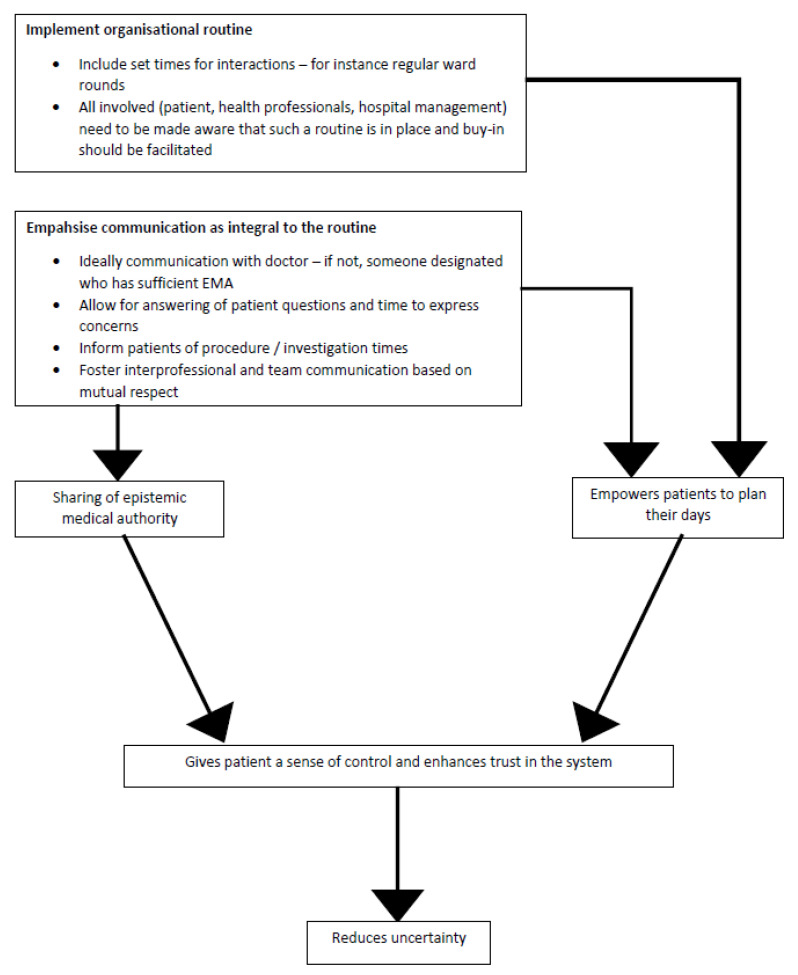
Organisational routine, communication and sharing of EMA.

Our findings confirm some of the in-patient experiences of oncology care as reported in the previous literature. They also suggest that broader issues of communication, organisational routine and epistemic authority mediate experiences of oncology care (Figure 2). When communication breaks down between health professionals and patients, or between team members, this can have significant implications for the quality of care provided and received. Such breakdowns may relate to an absence of organisational routine within the clinic space. If patients do not feel they are able to access information and their own expertise is not acknowledged, this may lead to feelings of dissatisfaction and ultimately complaints about the care received.

### 4.1. Organisational Routines

Organisational routines are repeated actions across regular intervals in which several interdependent role-players cooperate within certain rules and boundaries to bring about a specific outcome. Greenhalgh [11] describes numerous aspects that contribute to routine in health care settings, and she argues for the importance of routines in maintaining and improving the quality of care. Importantly, routines can alleviate uncertainty for patients as well as for health professionals.

In our setting, barriers to establishing effective organisational routines included communication breakdowns between patients and healthcare providers, a lack of predictability in interactions with doctors, limited access to information and diminished confidence in nurses. If patients do not feel they can access information and their expertise is not acknowledged, this may lead to feelings of uncertainty, dissatisfaction and lacking control.

Ironically, nurses, who are often at the frontline of patient management and arguably have the largest amount of patient contact, appear to be underutilised or disabled by the healthcare system as conveyors of information. In many settings, a deeply ingrained healthcare hierarchy as well as patient perceptions that medical expertise (epistemic authority) lies with medical rather than nursing staff, means that opportunities for communication and information sharing in interactions with nurses may be missed [12,13]. As suggested by our results, this is entrenched in perceptions amongst patients of nurses as a lesser source of epistemic authority than doctors.

Patients appear to experience a great sense of uncertainty during their inpatient journey, with a unique and complex set of psychological and physical challenges. On the one hand, oncology inpatients are expected to comply with routines that have been adopted by the institution, such as waking up for blood draws in the early hours of the morning so that blood results are available when ward rounds take place. On the other hand, between these events, hospital time is largely unstructured, exacerbating uncertainty. Oncology inpatients also struggle to initiate their own daily routines within this framework. A sense of certainty for inpatients could be achieved through robust organisational routines which emphasise some of the elements our study found to be lacking.

The presence of an organisational routine in itself does not seem sufficient to improve quality of care without a strong focus on effective communication and on building relationships between health professionals and patients and amongst health care team members [14]. For example, in the relationship between patients and doctors, routines such as regularly timed doctors’ visits must be rooted in regular communication, information sharing and an acknowledgement of expertise on both sides. Moreover, there is a need for greater dialogue between team members and the negotiation of organisational routines as part of a commitment to breaking down institutional hierarchies to promote patient-centred care.

### 4.2. Epistemic Medical Authority

The embedding of organisational routines into the healthcare setting requires a collective commitment to identifying the locus of Epistemic Medical Authority (EMA) in a healthcare interaction and facilitating its realisation. EMA is a theoretical component of the shared decision-making model [15]. According to Barnoy et al. (2012), EMA is “… a judgement of the extent to which someone possesses valid knowledge in a given domain” [16]. In today’s era of patient-centred care, the degree to which decision making is truly a joint effort depends on how the epistemic status of each role-player is viewed subjectively by the other role-players in the interaction.

EMA is a bidirectional concept, as it can be located both with patients and health professionals. Patient self-knowledge resides in the subjective domain formed by living and experiencing an illness. It is referred to as the ‘epistemics of experience’ [17]. The realm of medical expertise belonging to doctors is referred to as the ‘epistemics of expertise’ [18,19]. Ideally, the information located in the epistemics of experience and the epistemics of expertise should be recognised by all parties in order to facilitate optimal access to EMA.

The shared decision-making model requires input from patients. In order to fully participate in their care, patients need information upon which to deliberate and base decisions. For patients, this information typically resides with the locus of EMA, generally the doctor. As the locus of EMA, it follows that regular communication with said doctor is essential to transmit the information which patients deem most necessary. Patients experience heightened uncertainty when communication with the EMA is limited. With regular access to EMA, uncertainty for patients can be allayed, promoting a sense of control [15,16,17,18,19].

Our results suggest that the epistemics of experience may be well-developed in patients, but not always readily accepted by health professionals responsible for patient management, making patients feel uncertain. Patients also expressed a lack of access to information from the realm of the ‘epistemics of expertise’ [15,17], and they attributed a higher level of epistemic authority to doctors than to nurses. Interestingly, doctors and nurse managers in our sample also attributed lesser epistemics of expertise to nurses. The observation of this phenomenon by patients may account for a lack of patient confidence in nurses and also a lack of nurse self-confidence and empowerment.

It is this void in both accessing and sharing epistemology (which can only happen through communication) that may account for patients feeling uncertain in the study setting and explains the desire for control. As discussed previously, this could be facilitated through robust organisational routines which emphasise some of the elements that our study found to be lacking. These include good communication, managing expectations and allowing time for planning. However, in our setting, lack of uniformity in interactions with doctors, lack of information and lack of confidence in nurses were barriers to establishing routine or effectively eliciting information from the EMA.

In order for organisational routines to be effective, regular communication and interaction (ideally with one’s treating doctor) is vital. At this juncture, the epistemics of experience meets the epistemics of expertise. Routines must be rooted in communication and be structured around regularly timed doctors’ visits. They should be long enough to allow sufficient time for answering patients’ questions and addressing their concerns. Only in this manner can the epistemics of experience take its rightful place in the medical management trajectory, increasing the epistemic status of patients. Published studies suggest that this increase in EMA can reduce uncertainty [18]. Through the sharing of EMA, patients would be able to plan their days in hospital, and also hopefully into the future (Figure 2).

### 4.3. Clinical Implications

Based on our findings, we suggest that implementing robust organisational routines for oncology inpatients may be a good mechanism for allaying uncertainty and conferring a sense of control. These routines need to include a strong focus on improved communication amongst healthcare team members and with patients. Nursing staff, as the individuals with the most direct patient contact, could be instrumental in nurturing organisational routines to impact patient perceptions of care, but this needs to be done within a wider framework where nurses are seen as key members of the team and given the attendant responsibility within the institutional hierarchy. Nurses need to be seen as a locus of EMA, and placed as such through deliberate institutional policies.

## 5. Conclusions

Oncology inpatients who are hospitalised during their cancer treatment may feel they are not in control of their circumstances and unable to plan their days due to a lack of routine in the hospital setting. This lack of control seems to stem in part from an inability to have their experience of illness (epistemics of experience) recognised, combined with frustration in accessing health-related information from a source deemed to be reliable (epistemics of expertise). Improved communication, nested in an organisational routine that has the buy-in of patients and staff alike, could facilitate improved oncological care, decreased uncertainty and greater satisfaction with the quality of care received.

## 6. Study Limitations and Future Research

This study is based on a small sample at a single hospital in a particular geographic location and was conducted with a group of patients whose backgrounds were not culturally and linguistically diverse. This may affect the application of the findings in other contexts. Although our findings may not necessarily reflect the care experiences of patients and health professionals in other oncology settings, we believe there are some important clinical implications that emanate from this study.

Our study indicates the need for future studies to explore the perspectives of both patients and health professionals when it comes to examining experiences of care, in order to appreciate the demands that this complex illness and treatment context places on both patients and health professionals. It could be argued that the findings of our study are not necessarily unique to the experience of inpatient cancer care and may be applicable to other diseases, and future areas for enquiry in the area could include such work. A pragmatic, longitudinal implementation study centred around organisational routine and assessing its effectiveness would also be a compelling direction for future research.

## Figures and Tables

**Table 1:** Main themes identified in the data.

Theme	Patient Perceptions	Nurse Perceptions	Doctor Perceptions
**Expertise**	**Patient expertise** Patients physically experience their cancer. In this narrow sphere, they have unique insight into their particular disease → not always acknowledged by nurses and doctors.	**Nurse expertise questioned** Nurses feel their ‘side of the story’ is not always heard and fear retribution. Struggle to get their individual nursing expertise acknowledged by management and doctors.	**Nurse expertise doubted** Doctors not always sure that nurses know what they are doing.
**Information**	**Need for information** Patients expect and request info. This is not always forthcoming, and patients spend a lot of time waiting for info from doctors. Doctors and nurses provide contradictory information.	**Information uncertainties** Unsure what/how much information to give patients and struggle to get information from doctors. Worse when doctor cannot be contacted. Aware that information provided to patients may contradict that of the doctor.	**Sufficient information given** Doctors feel they are giving enough information to patients.
**Doctor** **Availability and team** **Communication**	**(Un)availability of doctors** Doctor ward rounds ad hoc, rushed and unplanned = frustrating for patients. See doctor as very important to get information but doctors often too rushed to listen and answer questions. Feel doctors provided more accurate information than nurses.	**(Un)availability of doctors off premises** Express trepidation about phoning doctors who are not on-site when advice/instruction is needed. Doctors are not always polite when contacted.	**Doctor availability OK** Doctors acknowledge that ward rounds rushed and ad hoc, with little routine. Done when doctors “have a minute”. Doctors feel that communication with patients sometimes rushed, but extensive and adequate for the majority of the time.
**Routine**	**Organisational routine** Patients want more information on what, why and when interventions (surgery, radiology) will happen. Overwhelming need to ‘plan their days’ and feel a sense of control.	**Challenges promoting** **routine** Nurses struggle to receive information about intervention times from radiology, theatre and ward doctors, and feel frustrated as they cannot provide this information to patients.	**Lack of organisational routine** Doctors acknowledge lack of structure for patients.
**In practice**	**Staff responsiveness** Nurses take a long time to answer the bell, which varies depending on shift/time of day. Patients feel they irritate nurses by ringing the bell. Handover is a particularly bad time.	**Roles and responsiveness**—Nurses aware that response times are slow. Whose job is it? Higher nurse ranks feel lower ranks should do it, yet lower ranks and CWs are not always sufficiently skilled to address issues patients raise when they ring the bell.	**Etiquette and the telephone** Nurse phone skills are seen as lacking, especially when contacting doctors at night.
**Psychology**	**Empathy and kindness** These are vital. Overall experience of patients who received empathy and kindness is much better than those who do not.	**Stress and distress in the workplace** “Heavy patients” with complex cancers and demanding families are difficult to manage. At times, nurses felt unsupported, they experienced moral distress and were inadequately trained to deal with these issues.	**Type of management** Stress and ‘heavy patients’ were acknowledged. Drs prefer to work with ‘active therapy’ patients rather than palliative. They do not feel trained to have difficult conversations with families.
**Work** **Relationships**	N/A	**Professional relationships** **(1) with doctors**: Variable, depends on the doctor. Some expressed good relationships, while others felt uneasy and that they were not always treated with professional respect. **(2) with nurses**: Lacking teamwork is seen as a problem. If working as a team, many problems could be addressed.	**Teamwork** A sense of isolation was expressed. Doctors felt a lack of teamwork with their medical peers and a lack of peer review. They expressed enthusiasm for more multi-disciplinary environment.
**Professional skills**	**Overall positive** Feel doctors are highly skilled and have medical competence comparable to international standards. Most feedback about nurses was also positive.	**Professional competence** Sometimes felt under-skilled in caring for complex cancer patients. Training has been very well received.	**Role and scope** Doctors felt they were often taking on role of counsellor to patient and family but did not feel trained to do this.

## Data Availability

The data that support the findings of this study are not publicly available due to privacy or ethical restrictions.
